# New Horizons in the Treatment of Corneal Endothelial Dysfunction

**DOI:** 10.1155/2021/6644114

**Published:** 2021-07-09

**Authors:** Carlos Rocha-de-Lossada, Rahul Rachwani-Anil, Davide Borroni, José-María Sánchez-González, Raquel Esteves-Marques, Fernando-Luis Soler-Ferrández, Jose-Antonio Gegúndez-Fernández, Vito Romano, Eitan Livny, Marina Rodríguez Calvo-de-Mora

**Affiliations:** ^1^Qvision Department of Ophthalmology, Hospital VITHAS Virgen Del Mar, Almería, Spain; ^2^Department of Ophthalmology, Hospital Virgen de Las Nieves, Granada, Spain; ^3^Department of Ophthalmology, Ceuta Medical Center, Ceuta, Spain; ^4^Department of Ophthalmology, Hospital Regional de Málaga, Málaga, Spain; ^5^Fondazione Banca Degli Occhi Del Veneto Onlus, Zelarino, Venezia, Italy; ^6^Department of Doctoral Studies, Riga Stradins University, Riga, Latvia; ^7^Department of Physics of Condensed Matter, Optics Area, University of Seville, Seville, Spain; ^8^Department of Ophthalmology, Tecnolaser Clinic Vision, Refractive Surgery Centre, Seville, Spain; ^9^Department of Ophthalmology, Hospital de Santa Maria, Centro Hospitalar Universitário Lisboa Norte, Lisboa, Portugal; ^10^Vision Sciences Study Center, CECV, Facultade de Medicina, Universidade de Lisboa, Lisboa, Portugal; ^11^Department of Ophthalmology, Innova Ocular Clinic Dr. Soler, Elche, Spain; ^12^Department of Ophthalmology, San Carlos Clinic Hospital, Madrid, Spain; ^13^Instituto Universitario, Universidad de Oviedo and Fundación de Investigación Oftalmológica, Oviedo, Spain; ^14^Department of Ophthalmology, Royal University Liverpool Hospital, Liverpool, UK; ^15^Department of Ophthalmology, Rabin Medical Center, Petah Tiqva, Israel; ^16^Department of Ophthalmology, Sackler Faculty of Medicine, Tel Aviv University, Tel Aviv, Israel

## Abstract

The treatment of corneal endothelial dysfunction has experienced a revolutionary change in the past decades with the emergence of endothelial keratoplasty techniques: descemet stripping automated endothelial keratoplasty (DSAEK) and descemet membrane endothelial keratoplasty (DMEK). Recently, new treatments such as cultivated endothelial cell therapy, Rho-kinase inhibitors (ROCK inhibitors), bioengineered grafts, and gene therapy have been described. These techniques represent new lines of treatment for endothelial dysfunction. Their advantages are to help address the shortage of quality endothelial tissue, decrease the complications associated with tissue rejection, and reduce the burden of postoperative care following transplantation. Although further randomized clinical trials are required to validate these findings and prove the long-term efficacy of the treatments, the positive outcomes in preliminary clinical studies are a stepping stone to a promising future. Our aim is to review the latest available alternatives and advancements to endothelial corneal transplant.

## 1. Introduction

### 1.1. The Evolution of Keratoplasty

Corneal endothelium is formed by a single layer of hexagonal cells that preserve corneal transparency by regulating the outflow of aqueous humor (AH) to the stroma through its barrier and pump mechanisms. It is supposed that corneal endothelial cells (CEC) have a limited regenerative capacity *in vivo* as they remain inactive in the G1 phase of the cellular cycle [1]. When there is a loss of CEC, the damage triggers a countervailing migration and an increase in the size (polymegathism) of the adjacent healthy CEC, resulting in a global decrease in endothelial cell density (ECD) in order to restore the single layer of CEC [1].

Fuchs Endothelial Dystrophy (FED) is a bilateral, sporadic, or autosomal dominant or corneal dystrophy that involves a progressive loss of CEC [2, 3]. Pseudophakic bullous keratopathy (PBK) is caused by an accelerated loss of CEC, mainly after cataract surgery though it is also described after other procedures [3]. Both entities are the most common indication for keratoplasty in the USA [3]. Over 100 years, penetrating keratoplasty (PK) has been the only surgical technique for the treatment of corneal diseases. In the past two decades, PK has been gradually replaced by lamellar keratoplasties for the treatment of endothelial disorders [4–6]. Descemet stripping automated endothelial keratoplasty (DSAEK) is an additive surgery as the donor graft includes the DM, endothelium, and a portion of stroma [7]. Descemet membrane endothelial keratoplasty (DMEK) was introduced later as a finer modification of endothelial keratoplasty (EK), and it comprises the transplantation of the DM and endothelium [5, 6]. DMEK has proven to attain better results in best-corrected visual acuity (BCVA) and a faster recovery compared to PK and DSAEK [6, 8–10]. Nevertheless, this technique has reported to have a longer learning curve than DSAEK, and a higher rate of postoperative graft detachment which is usually balanced after the learning curve [10–13]. The use of thinner grafts in DSAEK (<100 *µ*m), termed ultrathin DSAEK, shows better BCVA results compared to standard DSAEK, although it has not proven to be superior to DMEK in BCVA results nor in complication rates [13–17].

**Figure 1:**
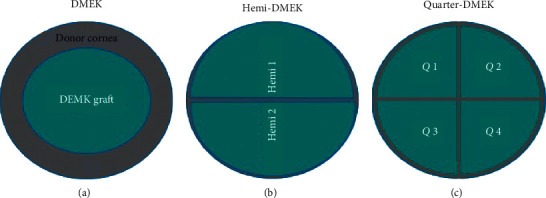
Comparison of graft diameter in DMEK (8.5 to 9.5 mm), hemi-DMEK (11-12 mm × 5-6 mm), and quarter-DMEK (6 mm × 5 mm) (based on the articles by Lam et al. and Müller et al. [19, 28]).

Recent innovations of DMEK are hemi-DMEK [18] and quarter-DMEK [19]. Hemi-DMEK consists of 12 mm long × 5 mm wide semilunar-shaped grafts, proving an equivalent surface and postoperative ECD of a standard round 8 mm DMEK graft [20]. Quarter-DMEK comprises 6 × 5 mm grafts shaped as a quarter of a circle and has proven an equivalent surface and postoperative ECD to a 6 mm DMEK graft [21] (Figure 1). Although it is not authorized in all countries, the possible advantage of these techniques is to provide higher availability of endothelial donor tissues [21, 22]. Both techniques proved similar postoperative BCVA results, although ECD was lower than a standard DMEK [23–25]. However, BCVA remained stable after three years in hemi-DMEK and after two years in quarter-DMEK procedures [23–25]. Both techniques, especially quarter-DMEK, could be reserved for cases of central FED and patients with different anterior chamber (AC) abnormalities, such as peripheral anterior synechiae or the presence of glaucoma valve implants.

**Figure 2:**
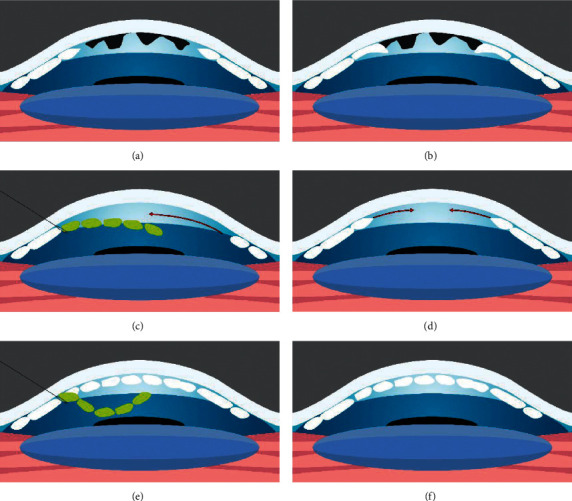
(a, b) Fuchs endothelial dystrophy disease with guttae protruding from the descemet membrane (DM). (a, c, e) The DMET technique. After descemetorhexis, the graft is inserted and fixated to the main corneal incision; the rest of it remains free-floating in the anterior chamber. (d) The DWEK/DSO technique in which a descemetorhexis is performed without further graft implantation. (b, d, f) The DMT technique in which descemetorhexis is performed and a DM graft devoid of endothelial cells is transplanted (based on the articles by Lam et al. and Bruinsma et al. [28, 89]).

Another technique termed descemet membrane endothelial transfer (DMET) was developed after observing corneal clearance despite subtotal graft detachment in patients operated for DSAEK or DMEK [26, 27]. In this procedure, the DMEK graft is introduced into the AC as a free-floating graft roll attached to the receptor cornea only by the main incision where the graft was introduced [28] (Figure 2). Interestingly, spontaneous clearance despite graft detachment only occurred in patients with FED and not in those with PBK [29]. Peripheral endothelium is relatively conserved in FED; hence, a migratory endothelial response of functioning peripheral cells could occur despite the graft not being completely attached [29]. Nevertheless, the cell regenerative capacity of FED patients might not be enough to guarantee permanent corneal transparency, as corneal decompensation six months after DMET has been reported [29].

## 2. Alternatives to Tissue Grafting

### 2.1. Descemetorhexis without Endothelial Keratoplasty (DWEK)/Descemet Stripping Only (DSO)

Some FED patients have reported corneal clearance by simply performing descemetorhexis intentionally or unintentionally [30]. The technique was named descemetorhexis without endothelial keratoplasty (DWEK) by Kaufman in 2018 [31] and was also called descemet stripping only (DSO) by Gorovoy [7] (Figure 2). However, the original idea was first described by Paufique in 1955 [32].

This technique is based on the assumption that the remaining peripheral CEC could migrate onto the denuded central stroma [30, 33, 34]. As mentioned priorly, CEC have a limited regenerative capacity *in vivo* [35–37]. Therefore, it is generally believed that endothelial wound healing occurs through cell migration rather than the proliferation of new cells [34]. However, stem cell markers (LGR5) have been identified in the posterior limbus near the trabecular meshwork [35–37], hence suggesting that some endothelial stem cells may be involved in endothelial wound repair [30, 34, 38].

A series of cases by Koenig, Bleyen et al., and Arbelaez et al. [30, 33, 39] described failure or inconclusive results of DSO after an 8 mm, 6–6.5 mm, and 6 mm descemetorhexis, respectively. It was hypothesized that the rough zone could have been somehow linked to the disfunction and/or the damage of endothelial cells due to surgical trauma [32, 39]. Corneal clearance has been reported after performing a smaller descemetorhexis (4 mm) in the following studies: Ioveno et al. [40], in four out of five cases; Borkar et al. [41], in 10 out of 13 eyes; and nine out of 12 in the series of Garcerant et al. [32]. Thus, it seems that DSO achieves better results when descemetorhexis is performed with a smaller size [32, 40, 41].

An increase in the descemetorhexis diameter from 4 to 6 mm requires more than double the surface area for the remaining endothelium to repopulate, whereas an 8 mm descemetorhexis requires a repopulation of four times the area of a 4 mm descemetorhexis [40]. Consequently, DSO would be better reserved for patients with central and nondecompensated FED, with a good peripheral CEC reservoir (over 1,000 cells/mm^2^), considering the relatively low postoperative central CEC count described [40–42]. Borkar et al. [41] reported that corneal transparency was achieved in different time periods after undergoing DSO. These periods were as follows: from after one to three months (fast responders), after six months (slow responders), and unsuccessful surgeries that required EK (no responders).

There is some disagreement among ophthalmologists whether a secondary EK performed after an unsuccessful DSO could achieve favorable results. Both Rao et al. and Moloney et al. [43, 44] reported positive outcomes. Therefore, DSO may not hinder the outcome of a secondary EK if necessary [32]. However, some authors, such as Arbelaez et al. [39], suggested that a subsequent DMEK graft may not easily adhere to areas that were stripped off and then repopulated with the endothelium, unless the repopulated endothelial cell layer is removed. Future prospective studies are required to confirm these findings.

Combination of DSO with cataract surgery does not seem to affect the results, hence being a viable option [31, 32, 40–42]. Borkar et al. [41] stated that approximately 75% of eyes that had combined DSO and cataract with IOL placement surgery showed corneal clearance and repopulation of the central endothelial mosaic by confocal microscopy.

However, the results of DSO are inconsistent as some studies have reported the failure of this technique in achieving corneal transparency [40, 45]. It is reasonable to suppose that surgery outcomes may depend on patients' innate features, possibly genetic, that involve CEC migration ability, anterior segment configuration, and surgery-related factors [32]. For instance, Davies et al. [45] stated that achieved corneal transparency time period after DSO in the fellow eye was observed to happen in the similar time period as the first eye, suggesting that patients' innate factors, such as growth factors in the AC, could be involved although they are yet to be defined [32, 45]. It is possible that differences in the number of trinucleotide repeated expansions in FED patients may affect the success or failure of DSO [41]. It is worth mentioning that an *in vitro* analysis of endothelial cell migration by Soh et al. [46] identified that younger ages and intact DM are important factors that may promote cell migration.

Soh et al. [46] found that CEC migrate more efficiently over a denuded but intact DM compared to bare stroma. Similarly, Garcerant et al. [32] described posterior stromal scarring in the edematous zone during the corneal clearance process in slow responders or nonresponders. Therefore, they assumed that surgical trauma of the stroma could induce an unpredictable healing response favoring fibrosis, hence recommending a surgical procedure that avoids stromal contact. They recommend using a peeling technique, to maximize both cell preservation and migration [32], as they observed an increased cell loss in techniques where constant pressure was applied during Descemet's scoring. This theory is supported by Davies et al. [45], who observed that DSO performed with a 360-degree scoring technique resulted in a visually significant stromal scarring, either from the scoring itself or from persistent edema. This group described that all failed cases in healing after DSO shared the 360-degree scoring technique followed by stripping. Nevertheless, all cases that underwent stripping by peeling without scoring cleared successfully [32, 45]. They proposed that manual stripping can result in an irregular DM border that promotes small DM detachments and edema [45]. Macsai and Shiloach [47] recommended attempting a smooth transition edge without any interruptions of subjacent stromal fibers by a slow and steady aspiration using the irrigation/aspiration handpiece connected to the phacoemulsification unit. The DM should be torn in a curvilinear fashion such as the capsulorhexis technique in cataract surgery.

Regarding postoperative visual quality, Garcerant et al. [32] had the following theory explaining irregular astigmatism despite corneal clearance [40]. First, they described central corneal thinning in cases that attained corneal clearance [32, 46]. It is known that any corneal procedure that leads to central corneal thinning may simulate a myopic ablation, and a small or off-centered optic zone may induce higher-order aberrations [32]. It is therefore hypothesized that off-centered descemetorhexis could act as an off-centered optical zone and be the cause of visual disturbances. Thus, it is highly recommended to attempt symmetry and to meticulously center the procedure [32]. Lastly, performing relaxing incisions in DM may possibly have an astigmatic effect [32].

Regarding BCVA, DSO has proven to be successful in some patients: Borkar et al. [41] reported BCVA between −0.12 and 0.00 LogMAR. Davies et al. [45] achieved corneal clearance in 14 (82.4%) eyes, with a corneal edema resolution meantime from 3.14 to 6.17 months. Out of the 14 eyes cleared, 13 eyes achieved a BCVA of 20/25.

Huang et al. [42] compared visual outcomes of 12 DSO with 15 DMEK cases in mild to moderate FED. Although meantime to achieve 20/40 vision was longer for DSO than DMEK cases (2.2 ± 2.8 weeks compared to 7.1 ± 2.7 weeks, respectively), they found no statistical differences in final BCVA with less rate of adverse events in the DSO group. Huang et al. [42] did not provide ECD comparison between the two groups. Therefore, their conclusion [42] of relatively similar results among both DSO and DMEK should be taken cautiously.

As a donor graft is not necessary, the short-term (graft detachment and postoperative elevated intraocular pressure (IOP) due to topical steroid treatment or air bubble placement) and long-term complications (rejection, glaucoma, secondary cataract, potential disease transmission, or infectious keratitis) are reduced. On the other hand, lower postoperative ECD has been reported following this technique [39].

Therefore, despite contradictory outcomes, it may be reasonable to include DSO as a potential technique to treat endothelial disorders, especially for the treatment of central FED. It would be useful in areas with difficult access to donor grafts, in personal circumstances that could force patients to refuse graft surgery or when side effects of this technique outweigh the benefits. Although longer follow-up studies are needed, a recent retrospective case report of a successful and stable 5-year, bilateral DSO [48] suggested stability in the short term.

### 2.2. Descemet Membrane Transplantation (DMT)

Primary descemetorhexis followed by acellular descemet membrane transplantation (DMT) [49] is a recently introduced technique for FED patients. Although donor tissue is required, no donor CEC are needed for DMT, which majorly increases the donor pool and decreases the risk of rejection. Similar to DSO, it seems to work better with smaller stripped areas that leave peripheral CEC intact (Figure 2).

## 3. ROCK Inhibitors

RhoA/Rho-kinase (ROCK) intracellular pathway plays a role in actin cytoskeleton regulation and actomyosin contractile forces [50, 51], as well as numerous cellular processes that include cell proliferation (especially cell cycle progression), migration, adhesion, rigidity, morphology, apoptosis, and extracellular matrix reorganization [35, 36, 50–53]. The effect of ROCK pathway signaling seems to be dependent on each type of cell.

ROCK signaling is involved in numerous pathologies such as vascular diseases, cancer, asthma, insulin resistance, renal insufficiency, osteoporosis, neuronal degenerative diseases, and glaucoma [35, 52]. Thus, ROCK inhibitors have been conceived as a therapeutic target for the treatment of several conditions [35, 52].

Regarding glaucoma, ROCK inhibitors alter trabecular meshwork configuration, increasing AH outflow through the trabecular pathway, hence decreasing IOP [43]. Two ROCK inhibitors have been approved for the treatment of ocular hypertension and glaucoma: ripasudil (Glanatec™) and netarsudil (Rhopressa™) [53].

### 3.1. ROCK Inhibitors and Corneal Endothelium

CEC have proliferative activity *in vitro*, implying that corneal endothelium could proliferate under appropriate conditions [36, 52, 53]. The latest evidence supports that ROCK inhibition stimulates *in vivo* CEC proliferation, as well as cellular migration and apoptosis suppression [35]. Therefore, ROCK signaling modulation could be a potential therapeutic target for the early phase of the corneal endothelial disease [35–37, 52–54].

### 3.2. Studies in Animals

Okumura et al. [55, 56] reported that ROCK inhibitor Y-27632 increased cellular proliferation *in vitro* of cultivated CEC in primates. Later on, both Koizumi et al. and Okumura et al. [54–56] from the Kinoshita group proved its use in *in vivo* corneal endothelial dysfunction models in rabbits [55, 56] and primates [52]. They demonstrated that topical Y-27632 improved ECD, corneal edema, wound size, and scarring of endothelial wounds. They also confirmed that CEC proliferation in rabbits increased in a dose-dependent pattern after the instillation of Y-27632.

### 3.3. Studies on Humans

The efficacy of Y-27632 for the treatment of central corneal edema caused by FED has recently been investigated [36, 53, 54]. Koizumi et al. [54] carried out a study observing a stable reduction in central corneal thickness in three out of four eyes after topical Y-27632 application six times a day for one week. Similarly, Okomura et al. [53] found a recovery of corneal transparency in eight patients after being treated with topical Y-27632. These findings suggest that topical Y-27632 could be clinically beneficial for patients with central corneal edema secondary to FED [36, 53].

In both the studies mentioned above [53, 54], there were four cases of diffuse edema related to PBK that did not show a decrease in corneal thickness or any improvement in BCVA, despite the treatment with Y-27632.

Consequently, these findings suggest that topical Y-27632 could be clinically beneficial for patients with central corneal edema caused by FED, with less evidence in PBK [53, 54].

### 3.4. ROCK Inhibitors Combined with DSO

DSO in combination with topical ROCK inhibitors could improve BCVA results and may obviate or delay EK, therefore optimizing endothelial graft donor availability. Endothelial restoration without donor tissue could reduce higher-order aberrations and dispersion that often reduce BCVA after EK caused by the donor-receptor interface, mainly in DSAEK [47, 57, 58]. Soh et al. [46] found that Y-27632 supplementation may counterbalance the negative effect of older age in CEC migration.

Koizumi et al. [36] were the first to report the resolution of corneal edema caused by FED with the combination of endothelial denudation by transcorneal freezing and topical ROCK inhibitors. Macsai and Shiloach [47] studied the use of ROCK inhibitors in patients with FED with a peripheral corneal reserve >1,000 cells/mm^2^ that underwent DSO. In this study, nine patients were treated with ripasudil after DSO and another nine patients only underwent DSO. The use of ripasudil resulted in a faster BCVA recovery, higher central ECD after a year of treatment, and a decrease of peripheral ECD loss. Patients in the control arm showed a reduction in peripheral ECD by 10% after one year of treatment. Interestingly, the treatment arm showed no significant differences in peripheral ECD compared to preoperative values. The fact that the group treated with ripasudil revealed a postoperative ECD equivalent to preoperative ECD supports the concept of peripheral endothelial cell proliferation and/or migration after combining DSO with ripasudil.

DSO combined with ripasudil could imply an economical saving for society, as it does not require donor tissue nor much postoperative care. Moreover, Davies [59] recently observed that netarsudil could be effective in achieving corneal clearance in different cases of endothelial dysfunction that may present in a daily cornea practice, such as iridocorneal endothelial syndrome, after an early PK graft failure and after a chronic PK graft failure. Likewise, this has recently been verified by Schlötzer-Schrehardt et al. [60] in a large database with an *ex vivo* FECD tissue culture model, where a single dose of ripasudil induced a significant upregulation of genes and proteins related to cell cycle progression, adhesion, and migration of the cellular matrix, as well as increasing the endothelial pump and barrier function up to 72 hours after instillation without inducing adverse phenotypic changes.

### 3.5. ROCK Inhibitors and Cell Therapy

Tissue engineering has been suggested as a novel therapy that could replace conventional corneal transplantation [61, 62]. There are two possible available strategies to transplant cultivated CEC in receptor corneas: scaffold-based and cell-based [61, 62]. Scaffold-based strategy is based on transplanting cultivated corneal endothelium on a vector plate in a similar procedure to EK [35, 63]. Okumura et al. and Koizumi et al. [53, 63] and other researchers [64–66] have cultivated CEC on specific substrates. Examples of substrates are amniotic membrane [67], DM, human anterior lens capsule [68, 69], and bioengineered matrices composed of compressed collagen [70], gelatin [71, 72], silk-fibroin, and a combination of biopolymers. Subsequently, the resulting CEC sheets have been transplanted in animal models observing corneal clearing. However, these sheets are composed of a fragile single layer of cells and its attachment to the receptor requires a relatively challenging surgical technique [35].

Cell-based strategy is based on injecting cultivated CEC into the AC in the form of cell suspension. Okomura et al. [62] defended that cellular injection has certain advantages. For instance, it is a simple, noninvasive, and easy to prepare procedure. The injected CEC in the AC would not spontaneously attach to the receptor corneal endothelial layer, but ROCK inhibitors are known to improve the adhesion of CEC to a substrate [55]. This led researchers to pioneer animal experiments that proved the safety and efficacy of cultivated CEC injections in combination with ROCK inhibitors [35–37].

**Figure 3:**
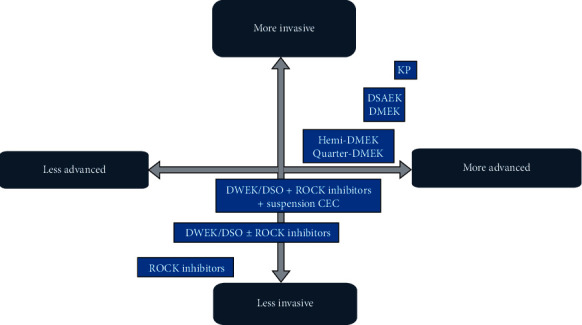
Schematic images of cultivated endothelial corneal cells (CEC) injected in the anterior chamber (AC) therapy. (a) CEC injected with a ROCK inhibitor in the AC; (b) prone position to help in the adherence of the cultivated CEC to the recipient stroma; (c) prone position should be maintained for three hours postoperatively; and (d) regeneration of the corneal endothelium by the injected CEC (based on the article by Okumura et al. [62]).

Kinoshita et al. [73] carried out a study on humans with a two-year follow-up. They included 11 patients, seven with FED and the rest with bullous keratopathy (BK) of various causes. A mechanic 8 mm descemetorhexis followed by an injection of cultivated CEC in combination with ROCK inhibitor Y-27632 was performed. After the procedure, the patients rested in a prone position for three hours (Figure 3). After six months, ECD >500 cells/mm^2^ was observed in all patients, and 10 out of 11 had an ECD >1,000 cells/mm^2^. Regarding visual outcomes, nine out of 11 showed a BCVA equal to or higher than 0.3 LogMAR. Furthermore, 10 out of 11 patients revealed a central corneal thickness <630 *μ*m. Two years after the procedure, all the corneas remained transparent, with an average ECD of 1,534 cells/mm^2^, and nine out of 11 patients had a BCVA equal or higher than 0.1 LogMAR.

**Figure 4:**
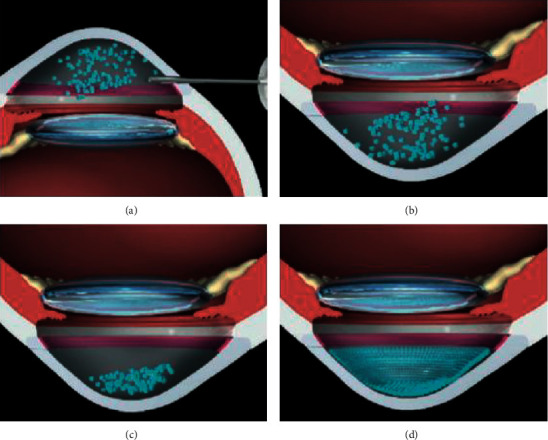
Future strategies for the treatment of endothelial diseases, from less invasive treatments to more invasive ones (based on the article by Okumura et al. [35]).

The authors hypothesized a few concerns, namely, what happened to the CEC that did not attach to the receptor endothelium and whether it could obstruct the trabecular meshwork or lead to iris adhesions. Another concern was that CEC could pass onto the systemic circulation and could potentially cause tumor development. However, according to the latest evidence, ROCK inhibitors and cell therapy can effectively be used in both FED and BK patients with optimal results (Figure 4).

Other substances that are currently being investigated for the treatment of endothelial diseases are antioxidants, such as N-acetylcysteine, coenzyme Q-10, sulforaphane [74], RTA-408 [75], and fibroblast growth factors, such as FGF-1 and bioengineered eFGF synthesized by Trefoil™.

## 4. Gene Therapy

Two types of gene therapy could play an important role in corneal diseases: antisense oligonucleotides (ASO) and prokaryotic clustered regularly interspaced palindromic repeats (CRISPR) [76, 77].

An ASO molecule consists of a small sequence of nucleotide fragments complementary to a specific gene sequence (messenger RNA, mRNA). In antisense therapy, base pairing between the ASO molecule and mRNA inhibit gene translation hence disabling protein synthesis. The CRISPR are a defense mechanism against virus present in bacteria and archaea. They consist of a palindromic short sequence DNA, originated from the virus that has previously infected these bacteria. These DNA loci are usually associated with Cas genes that code a type of nuclease (enzymes that can split DNA). CRISPR spacers recognize specific sequences and guide Cas nuclease to split and degrade exogenous genic elements [77]. Thus, when a virus attacks a determined bacterium, it interacts with the Cas protein complex bound to the RNA produced by the CRISPR sequence. Then, the viral genetic material gets inactivated, degraded, modified, and integrated in the CRISPR sequence. Ultimately, the defense will be more effective in case of a future contact of the bacteria or its descendants with the affected virus.

The CRISPR/Cas9 system could be used to edit and regulate the genome [78]. A RNA molecule can be designed and inserted in the nucleus, where it recognizes the exact genome location that the Cas9 enzyme must split. Later, a second mechanism allows the split DNA to be repaired, embodying the correct genetic sequence in the exact original site of splitting [78].

Although FED is a heterogenous genetical disease, a major number of patients, especially Caucasians, possess a pathological trinucleotide expansion sequence (typically, cytosine-thymine-guanin (CTG) in the TCF4 gene located in chromosome 18q21) [76, 79]. ASO molecules targeting specific trinucleotide expansion mRNA and CRISPR/Cas9 systems designed to bind to DNA trinucleotide repeated sequences may interrupt these mRNA anomalous repetitions that cause some subtypes of FED, especially in cases of intermediate and short anomalous lengths [76, 80, 81].

Koenig [30] suggested that RNA toxicity contributes to the pathogenesis of FED. Changes in the endothelial barrier function, a known event in the development of FED, were identified as a key biological process influenced by the misplacing events. Moreover, anomalous DNA segments may possibly be directly excised by endonucleases, such as transcription activator-like effector nucleases (TALENS) [82]. These findings support that gene therapy could be effective in treating the genetic defects responsible for some types of FED, therefore changing the phenotype.

Recent studies have managed to administer transcription activators Cas9 molecules in vivo in CEC in rats, stimulating corneal endothelial proliferation and the restoration of normal endothelium after corneal cryotherapy. The latest research suggests that this technique could work on humans by adding additional improvements [83–86].

## 5. Mechanic Artificial Endothelium

**Figure 5:**
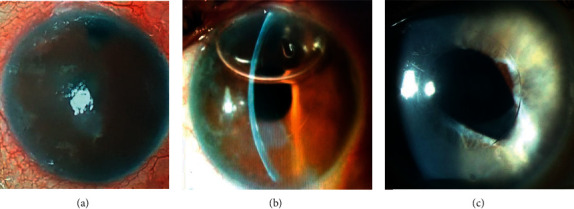
EndoArt® device in the first-in-human trial: (a) corneal edema prior to implantation. (b) The same eye on the first postoperative day. Note the air bubble at the AC that works as a tamponade agent. (c) Another eye several weeks following implantation. The central area corresponding to the implant zone is transparent, whereas the periphery outside the implant borders is edematous.

Endothelial dysfunction is manifested by corneal edema caused by endothelial pump malfunction. EndoArt® is a flexible silicon sheet covered with an adhesive substance, that is inserted into the AC and attached to the posterior surface of the cornea by air/gas pneumopexy, similar to a DMEK graft. This silicon sheet prevents the passive inflow of electrolytes and water into the cornea while allowing water evaporation from the corneal surface. Since this is a relatively new concept and device, there are no relevant peer-reviewed studies yet. However, the first experiments in humans after several years of animal studies were recently published in international meetings, showing promising results [87, 88] (Figure 5). This approach may be interesting in patients that cannot undergo EK, as a bridging procedure from diagnosis until EK is available, or even as a substitute to EK altogether. Nevertheless, a prospective, long-term study is needed to verify the promising preliminary results.

## 6. Conclusion

In the last decades, we have witnessed a true revolution in the treatment of corneal endothelial dysfunction. We have gone from penetrating keratoplasty as a sole therapy for all the corneal diseases, regardless of its origin and localization, to the great advancement that endothelial keratoplasty (EK) has supposed, being descemet stripping automated endothelial keratoplasty (DSAEK) and descemet membrane endothelial keratoplasty (DMEK) its two most exalted examples. The tenacious concept that corneal endothelial cells (CEC) cannot proliferate in vivo has been surpassed in recent years with research findings supporting that peripheral CEC possesses stem cell features. Similarly, many authors have proven that it is technically possible to cultivate and transplant CEC in both animals and humans.

Currently, we are witnessing the development of new techniques and therapies that try to reduce complications derived from EK: descemet stripping only (DSO), ROCK inhibitors, cellular therapy, bioengineered grafts, gene therapy, endothelial regeneration, and artificial endothelial substitutes. These procedures offer a new perspective in the treatment of endothelial dysfunction. Moreover, they contribute to mitigating the scarcity of quality endothelial donor tissue and decreasing the complications derived from the immune rejection of the donor graft, as well as reducing the use of steroid treatment. Although additional randomized prospective peer-reviewed trials are necessary to validate the findings and to confirm the effectiveness and safety of these procedures, the positive results in preliminary clinical studies predict a promising future.
